# What reward does a child prefer for behaving well at the dentist?

**DOI:** 10.1038/bdjopen.2017.18

**Published:** 2017-09-08

**Authors:** James Coxon, Marie Therese Hosey, Jonathon Timothy Newton

**Affiliations:** 1 Porth Dental Teaching Unit, Porth, UK; 2 Department of Paediatric Dentistry, King’s College London Dental Institute, London, UK; 3 Unit of Social and Behavioural Sciences, King’s College London, London, UK

## Abstract

**Background:**

Paediatric dentists often report using positive reinforcement to encourage their young patients to show co-operative behaviour. For effective reinforcement to take place the reward should be salient to the individual. To date, there is little research into what reward a young patient will choose when attending the dentist.

**Aim:**

To identify what reward children between the age of 4–8 years will choose when attending the dentist, and to determine the extent of agreement between children and caregivers in reward choice.

**Method:**

Observational study. Fifty-two children from different age groups (4–5 years, 6–7 years and 8 years) attending a primary-care dental clinic were asked to choose between a range of different rewards. The caregiver attending with them was also asked to anticipate the child’s preferred choice.

**Results:**

There was no clear favourite reward for children from both genders and different age group. However, no child chose the ‘sticker’ reward that is traditionally given out at the dentist. Overall carers agreed with the child’s choice of toy on 18 occasions (34.6%), but there were significant differences across the age groups with carers of older children showing less agreement

**Conclusion:**

To ensure that rewards are salient, children should be given a choice of rewards when attending the dental clinic. Parents ability to predict their child’s preferred rewards decreases as the child ages.

**Clinical relevance:**

A child’s motivation to co-operate during dental treatment can be increased by offering a range of rewards. Asking children to choose their reward from a limited range will increase the saliency of the reward for the child.

## Introduction

Paediatric patients frequently show undesirable behaviour that impacts on the ability of the dentist to treat them effectively,^1^ and dentists working with children are often required to instigate interventions to decrease the likelihood of these behaviours occurring, using the basic principles of behavioural psychology. These non-pharmacological techniques are widely supported throughout the international paediatric dental field.^2,3^ Positive reinforcement is one of the main behavioural modification methods in current use today.^4^

To allow positive reinforcement to be delivered effectively, the reward should be salient to the individual.^5^ However, there has been little formal research reporting what reward a child would prefer when attending the dentist. Experience suggests that most dental clinics in primary care use stickers as a reward. However, previous research has suggested that dentists’ knowledge of the principles underlying behavioural technqiues including the use of reinforcement is moderate, at best,^4,6^ Humza Bin Saeed *et al*. 2012 and so it is possible that the use of stickers as a rewards may not be an optimal strategy.

This study sought to research what reward children between the age of 4–8 years would choose as a reward for successful completion of treatment when attending the dentist, and to determine the extent of agreement between children and caregivers in reward choice.

## Method

Ethical approval was granted by the NRES Committee London—City Road & Hampstead (14/LO/0377). As the burden of participation was small, potential participants were informed of the study and asked to consent on the day of their attendance at the clinic, rather than before appointment. A patient information leaflet for both the child and the adult was given and written assent/consent taken.

A consecutive series of 52 children attending the Chief Investigator's dental surgery were shown 10 different small ‘rewards’ comprising toys and stickers (each costing less than £2.50 per unit, average cost per unit £1.53, 2016 prices). The rewards shown were different for each age group (4–5 years, 6–7 years and 8 years). The child was asked to indicate which of the 10 objects they would most like to be given after their dental visit. The child's response was then recorded and the child was given the object of their choice after the dental visit, regardless of their behaviour. Independently the child’s parent or caregiver was asked to indicate which of the items they believe the child would choose (without knowing which the child chose).

For each adult-child pair the following demographic data were collected:

- Gender of child.
- Age of child.
- Gender of caregiver.
- Relationship of caregiver to child.

The analysis of the data comprised:

1. A frequency count of token choice by child and by caregiver.
2. Calculation of the degree of agreement in object selection by parent and child.

## Results

Table 1 shows the number of children in each age group by gender. The difference in the gender distribution of the age groups was not significant. *χ*^2^=4.54, *P*=0.104.

### Reward choices

Table 2 shows the toy choices made by children and their primary caregivers broken down into the three age groups.

### Agreement between child and carer in toy choice

Overall carers agreed with the child’s choice of toy on 18 occasions (34.6%), but there were significant differences across the age groups with carers of older children showing less agreement (see Table 3, *χ*^2^=11.63, *P*=0.003). Gender of the child had no effect on the degree of agreement (Table 4, *χ*^2^=0.03, *P*=0.56).

## Discussion

This study demonstrated that there was no clear favourite reward for each age group and each gender. Therefore, to ensure that the reward is salient, children need to be presented with a wide range of choices. In addition, novel rewards may be more salient,^5^ which further suggests children should have a wide range of reward choices.

It may be argued that giving out rewards will leave a financial burden on the dental practice. However, the rewards chosen were all low cost, particularly in comparison to the cost of clinical time. When the child is well motivated to be co-operative, clinical time will be saved and treatment costs decreased.

It is unclear why caregivers of the older children were less likely able to anticipate what reward their child would choose. One hypothesis may be that parents of older children often have less contact with their child and are less aware of their likes and dislikes.

Further research is needed to highlight the benefits of using positive reinforcement techniques to gain co-operation in these age groups. Although salience of the reward affects the efficacy of reinforcement techniques, timing of the reward also has a measurable effect.^7^ Further work is needed to establish the effect of salient and timely reinforcement on the co-operation of paediatric patients.

## Figures and Tables

**Table 1:** Gender of child participants by age group

	*Male*	*Female*
4–5 Years	13 (62%)	8 (38%)
6–7 Years	7 (41%)	10 (59%)
8 Years	11 (79%)	3 (21%)

**Table 2:** Reward choices shown broken down by age and child/carer

	*Child choice*	*Carer choice*
*Age 4–5 years*		
Sticker choice 1	0	0
Sticker choice 2	0	0
Teeth	1 (5%)	0
Finger tattoo	1 (5%)	2 (10%)
Bubbles	7 (33%)	7 (33%)
Princess badges	5 (24%)	4 (19%)
Princess figures	2 (10%)	4 (19%)
Butterfly glider	0	0
Pirate whistle	1 (5%)	1 (5%)
Dino glider	4 (19%)	3 (14%)
		
*Age 6–7 years*		
Sticker Choice 1	0	0
Sticker Choice 2	0	0
Teeth	0	0
Egg	3 (18%)	2 (12%)
Despicable Me stationery	0	2 (12%)
Disney button badge	1 (6%)	4 (24%)
Highlighter	8 (46%)	3 (18%)
Alien sling shot	3 (18%)	2 (12%)
Dino glider	0	1 (6%)
Mini markers	2 (12%)	3 (18%)
		
*Age 8 years*		
Sticker choice 1	0	0
Sticker choice 2	0	0
Despicable Me stationery	2 (14%)	5 (36%)
Dino egg	3 (21%)	3 (21%)
Magic set	0	1 (7%)
Wooden horse	0	0
Cat’s cradle	2 (14%)	0
Plane model	0	1 (7%)
Marvel figure	5 (36%)	3 (21%)
Spy pen	2 (14%)	1 (7%)

**Table 3:** Extent of agreement between child and carer on toy choice, shown by age group

	*No agreement*	*Agreement*
4–5 Years	8 (32%)	13 (68%)
6–7 Years	14 (82%)	3 (18%)
8 Years	12 (86%)	2 (14%)

*χ*^2^=11.63, *P*=0.003.

**Table 4:** Extent of agreement between child and carer on toy choice, shown by gender of child

	*No agreement*	*Agreement*
Male	20 (64%)	11 (36%)
Female	14 (67%)	7 (33%)

*χ*^2^=0.03, *P*=0.56.
